# Non-Invasive Clinical Parameters for the Prediction of Urodynamic Bladder Outlet Obstruction: Analysis Using Causal Bayesian Networks

**DOI:** 10.1371/journal.pone.0113131

**Published:** 2014-11-14

**Authors:** Myong Kim, Abhilash Cheeti, Changwon Yoo, Minsoo Choo, Jae-Seung Paick, Seung-June Oh

**Affiliations:** 1 Department of Urology, Seoul National University Hospital, Seoul, Korea; 2 Department of Computer Science, School of Computing and Information Sciences, Florida International University, Miami, FL, United States of America; 3 Department of Biostatistics, Robert Stempel College of Public health & Social Work, Florida International University, Miami, FL, United States of America; Politecnico di Torino, Italy

## Abstract

**Purpose:**

To identify non-invasive clinical parameters to predict urodynamic bladder outlet obstruction (BOO) in patients with benign prostatic hyperplasia (BPH) using causal Bayesian networks (CBN).

**Subjects and Methods:**

From October 2004 to August 2013, 1,381 eligible BPH patients with complete data were selected for analysis. The following clinical variables were considered: age, total prostate volume (TPV), transition zone volume (TZV), prostate specific antigen (PSA), maximum flow rate (Qmax), and post-void residual volume (PVR) on uroflowmetry, and International Prostate Symptom Score (IPSS). Among these variables, the independent predictors of BOO were selected using the CBN model. The predictive performance of the CBN model using the selected variables was verified through a logistic regression (LR) model with the same dataset.

**Results:**

Mean age, TPV, and IPSS were 6.2 (±7.3, SD) years, 48.5 (±25.9) ml, and 17.9 (±7.9), respectively. The mean BOO index was 35.1 (±25.2) and 477 patients (34.5%) had urodynamic BOO (BOO index ≥40). By using the CBN model, we identified TPV, Qmax, and PVR as independent predictors of BOO. With these three variables, the BOO prediction accuracy was 73.5%. The LR model showed a similar accuracy (77.0%). However, the area under the receiver operating characteristic curve of the CBN model was statistically smaller than that of the LR model (0.772 vs. 0.798, p = 0.020).

**Conclusions:**

Our study demonstrated that TPV, Qmax, and PVR are independent predictors of urodynamic BOO.

## Introduction

Urodynamic study (UDS) is considered the gold standard for clinical assessment of bladder outlet obstruction (BOO) in patients with benign prostatic hyperplasia (BPH) [1]. Patients with urodynamic BOO show higher efficacy after transurethral surgery [2]. In this respect, BOO is helpful in stratifying BPH patients eligible for surgical treatment. However, UDS has significant limitations in terms of invasiveness, cost, and morbidity [3].

Numerous attempts have been made to substitute non-invasive clinical parameters for UDS to predict BOO; however, individual variables, including symptom score [4], prostate specific antigen (PSA) level [5], free uroflowmetry (UFM) [6], post-void residual (PVR) urine volume [7], and prostate size [8], have shown a poor to weak correlation with BOO.

To improve prediction ability, combinations of non-invasive clinical parameters have been investigated to predict BOO [9]–[17]. The statistical methods used for combinations were diverse from the cumulative scoring system [9], to the construction of a formula by logistic regression analysis [10]–[13], to the artificial neural network (ANN) models [14]–[17]. However, these attempts had limited predictive performance. Moreover, the need to use numerous clinical parameters makes clinical application difficult. Furthermore, some predictive models [14]–[17] could not explain which variables are comparatively important for BOO owing to their ‘black box’ nature [18].

Causal Bayesian networks (CBN) have emerged as an advanced alternative to conventional statistical models in the medical field [19]. The benefit of this model is that it can visualize the interaction of causes and rule out indirect causes of events [20]. Hence, we aimed to identify non-invasive clinical parameters to predict BOO using a CBN model. To the best of our knowledge, this study is the first to test CBN model for BOO prediction.

## Materials and Methods

### I. Data collection

The Institutional Review Board of Seoul National University Hospital (SNUH) approved the protocol of this study. A database of 2,492 patients that were older than 45 and that had lower urinary tract symptoms (LUTS) was created from records dated between October 2004 and August 2013. The data were retrieved from the urodynamic database registry and Electronic Medical Records System of SNUH. All information was anonymised and de-identified prior to analysis. Patients with a history of previous genitourinary surgery, pelvic radiation therapy, urinary tract infection, urethral stricture, interstitial cystitis, and neuropathy suggesting neurogenic bladder or incomplete evaluations were excluded. Thus, after excluding 1,111 such patients (44.6%), the data from 1,381 patients were analyzed.

Clinical parameters of the subjects, including history, physical examination, International Prostatic Symptom Score (IPSS) [21], UFM, PVR, PSA, prostate volume (PV) measured by transrectal ultrasonography, and UDS results were retrieved. UFM (Flowmaster, Medical Measurement System, Enschede, Netherlands) results were obtained as free flow, whenever voided volume was less than 120 ml, and fails were repeated. PVR was measured after UFM using an ultrasound bladder scanner (BladderScan BVI 3000, Verathon Inc., WA, USA). All UDS were performed using a multichannel video system (UD-2000, Medical Measurement System) according to International Continence Society (ICS) recommendations [22]. The BOO index, which is equal to detrusor pressure at maximal flow rate (PdetQmax)−2×maximal flow rate (Qmax), was used to determine BOO [23]. Patients with BOO Index ≥40 were considered as obstructed.

### II. Database characteristics

The patient demographics characteristics are shown in Table 1. The mean age of patients was 66.2 (±7.3, SD) years. The TPV and PSA were 48.5 (±25.9) ml and 2.71 (±3.53) ng/ml, respectively. The IPSS-total, IPSS-storage, IPSS-emptying, and IPSS-QoL were 17.9 (±7.9), 7.1 (±3.5), 10.8 (±5.5), and 3.9 (±1.2), respectively. The Mean BOO index was 35.1 (±25.2), and 477 patients (34.5%) were classified as having BOO.

**Table 1:** Characteristics of 1,381 patients.

	Total subjects (N = 1381)
Age (years)	66.2±7.3
Prostate volume (ml)	
Total prostate volume	48.5±25.9
Transitional zone volume	24.1±22.4
Prostate specific antigen (ng/ml)	2.71±3.53
International Prostatic Symptom Score (IPSS)	
IPSS-total	17.9±7.9
IPSS-storage	7.1±3.5
IPSS-emptying	10.8±5.5
IPSS-quality of life	3.9±1.2
Uroflowmetry parameters	
Maximum flow rate (ml/sec)	11.6±4.9
Post-void residual volume (ml)	58.1±77.8
Urodynamic study parameters	
Maximal urethral closure pressure (cmH_2_O)	74.3±26.8
Functional urethral length (mm)	72.0±20.6
First desire (ml)	203.0±90.1
Normal desire (ml)	284.9±108.2
Strong desire (ml)	371.7±108.3
Compliance (ml/cmH_2_O)	67.3±50.8
PdetQmax (cmH_2_O)	52.6±21.7
Opening pressure (cmH_2_O)	54.3±25.8
Bladder outlet obstruction index	35.1±25.2

PdetQmax, detrusor pressure at maximum flow rate.

### III. Statistical methods for BOO prediction

To predict BOO, the following two statistical methods were applied.

1. **Logistic regression (LR) analysis.** A backward stepwise regression analysis [24] was utilized. Age, total prostate volume (TPV), transition zone volume (TZV), PSA, Qmax, PVR and IPSS were entered into LR model as variables for BOO prediction. Relative risk (Exp(β)) of BOO was calculated, with each non-invasive parameter increasing by one unit.
2. **Causal Bayesian networks (CBN).** If event A causes events B and C, and these events directly influence event D, the probability of event D depends on each of the possible values of events B and C. The probability of event D can be expressed in the equation, P (*event D| event B, event C*). In that case, events A and D are in the causal Markov condition [25]. This means that event A is not a direct cause of event D. If the probabilities of direct causes (events B and C) are conditioned, event A does not influence the probability of event D. The causal Markov condition can be visually identified in a CBN, which has a relationship of two or more degrees between nodes. Considering that events A and D have a two degree relationship, we can easily infer that these two events are conditionally independent.

The causal Markov condition permits the joint distribution of the n variables in a CBN to be factored as in the following equation:
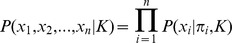
where *xi* denotes a state of variable Xi, *πi* denotes a joint state of the parents of Xi, and *K* denotes background knowledge [20].

### IV. Identification and verification of the independent parameters

CBN was applied to identify the independent predictors of BOO. The causal relationships and their interactions were visualized by established CBN. The variables only directly linked to BOO were selected as the independent predictors. The weights of each selected variable were estimated using the Spearman's correlation test. The accuracy of BOO prediction model using the selected variables was compared with that of the LR model. To compare the predictive performance, the comparison of receiver operating characteristic (ROC) curves by DeLong et al. [26] was applied.

A p-value <0.05 was considered significant. A CBN model to predict BOO was established using the Banjo version 2.2.0 software (Duke University, Durham, NC, USA; non-commercially available at: http://www.cs.duke.edu/~amink/software/banjo/). Highlights of the settings are limiting the number of parents to five and running the analysis for up to 6 hours (the Banjo setup file is presented in Appendix S1). The commercial statistical program package SPSS version 18.0 (Chicago, IL, USA) was used for LR, Spearman correlation, and other descriptive statistical analyses. MedCalc version 12.4.0 (Ostend, Belgium) was applied for the comparison of ROC curves.

## Results

### Identification of non-invasive BOO predictors using CBN

**Figure 1:**
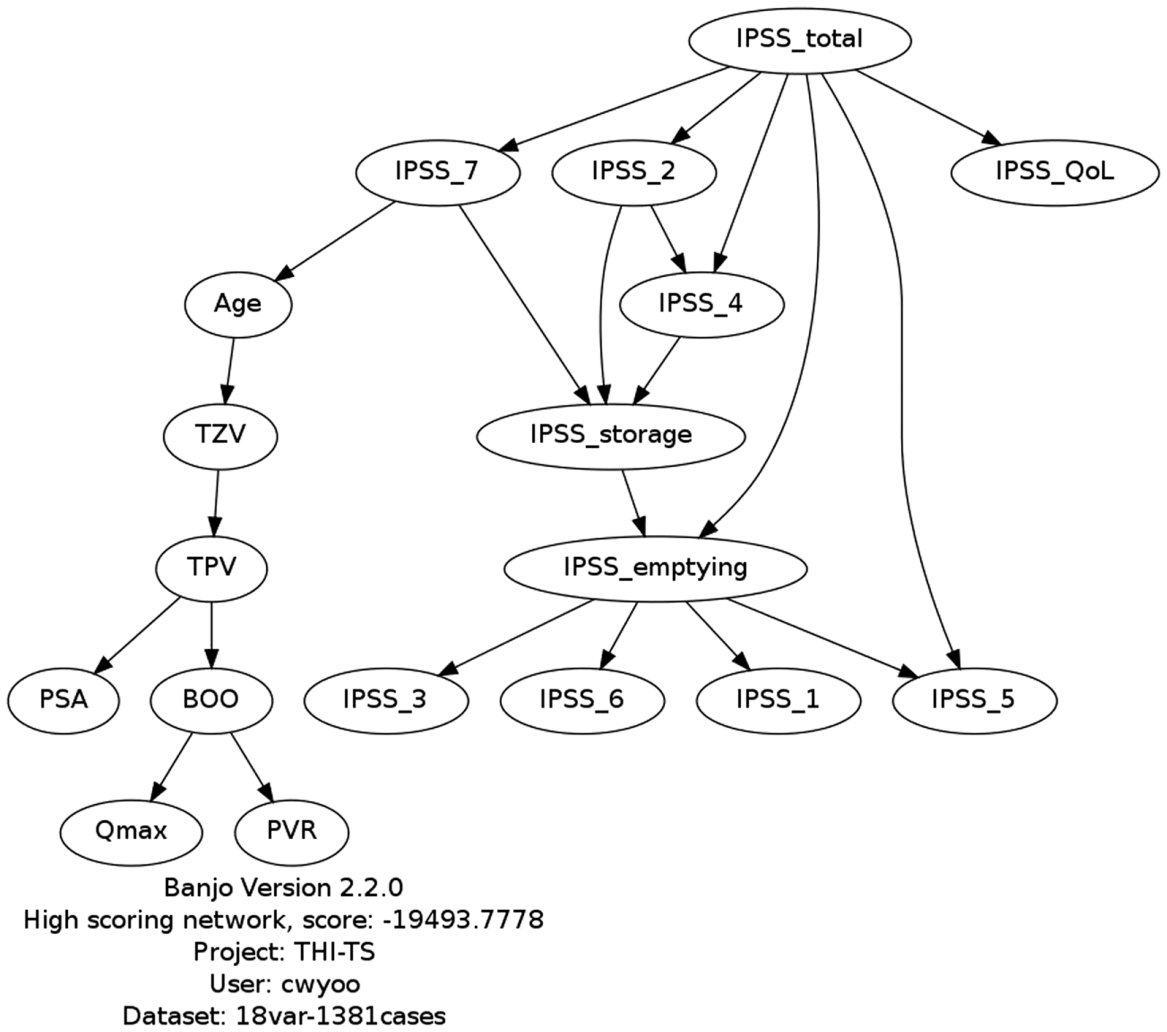
Causal Bayesian network model for bladder outlet obstruction. TPV, total prostate volume; TZV, transitional zone volume; PSA, prostatic specific antigen; BOO, bladder outlet obstruction; Qmax, maximum flow rate; PVR, post-void residual volume; IPSS, International Prostate Symptom Score.

Based on the BPH patient data, the best network structure was selected/learned using the CBN model (Fig. 1). TPV, Qmax, and PVR exhibited direct relationships with BOO. Therefore, those three variables were selected as non-invasive independent predictors of BOO. The correlation coefficient was the highest for TPV (R = 0.391 p<0.001), followed by Qmax (R = −0.253, p<0.001) and PVR (R = 0.214, p<0.001).

### Verification of BOO prediction

Sensitivity, specificity, and accuracy of BOO predictions with the aforementioned three variables by CBN were 51.4%, 85.2%, and 73.5%, respectively (Table 2). In the LR model, age (Exp(β) = 0.981, p = 0.046), Qmax (Exp(β) = 0.890, p<0.001), PVR (Exp(β) = 1.003, p<0.001), TPV (Exp(β) = 1.014, p = 0.049), TZV (Exp(β) = 1.039, p<0.001), PSA (Exp(β) = 1.051, p = 0.039) IPSS item 2 (frequency) (Exp(β) = 0.866, p = 0.007), and IPSS item 4 (Urgency) (Exp(β) = 1.227, p<0.001) were selected as significant predictive variables. In the LR model, the sensitivity, specificity, and accuracy were 51.6%, 90.4%, and 77.0%, respectively.

**Table 2:** Predictive value of two predictive models for bladder outlet obstruction.

	Predicted BOO		Urodynamic BOO		Total	Sensitivity	Specificity	Accuracy
			(+)	(−)				
LR model	Total (N = 1381)	(+)	246	87	333	246/477 (51.6%)	817/904 (90.4%)	(246+817)/1381 (77.0%)
		(−)	231	817	1048			
		Total	477	904	1381			
CBN model	Total (N = 1381)	(+)	245	134	379	245/477 (51.4%)	770/904 (85.2%)	(245+770)/1381 (73.5%)
		(−)	232	770	1002			
		Total	477	904	1381			

LR, logistic regression; ANN, artificial neural networks; CBN, causal Bayesian networks; BOO, bladder outlet obstruction.

To compare the predictive power of the three selected non-invasive clinical parameters, a comparison of ROC curves was performed. The area under ROC curve (AUROC) of CBN and the LR models were 0.772 and 0.798, respectively (p = 0.020; figures not presented).

## Discussion

Because individual variables have a very low correlation with BOO, many researchers have built statistical prediction methods that combine multiple variables [9]–[13]. For this purpose, they have used diverse variables, including Qmax, PVR, IPSS, PSA, and PV. However, no one has established a specific independent predictor of BOO [9]–[13]. Some differences in detailed variables have been suggested for prediction models. Moreover, the number of variables used in these predictions is too many to be feasible for real-life practice with BPH patients.

Previous studies seeking to identify non-invasive predictors of BOO have encountered two major difficulties. The first is the non-linear relationship between the variables. Among the single non-processed variables, prostate size seems to be one of the most highly correlating variables with BOO (R range: 0.28–0.32, p<0.001) [8], [27]. However, Eckhardt et al. [27] have found that mean TPV decreased at the Schäfer grade of 5 and 6, contrary to general expectations. These non-linear conditions occur commonly in clinical medicine. The second difficulty stems from the fact that some clinical parameters have a co-variability, fiu., some clinical parameters interact with each other [19], so that the established model is capable of overestimating or underestimating the predictive power. Bell et al. [28] reported that increased PVR occurs in BOO patients. However, Eckhard et al. [27] pointed out that larger PVR may reflect detrusor underactivity rather than BOO. Yet, Kranse et al. [7] supported the findings that BOO and detrusor underactivity commonly cause a higher PVR.

ANN models are expected to be able to detect non-linear relationships and interactions between predictor variables. Sonke et al. [14] proposed the first ANN model for BOO prediction using 1903 patients. IPSS, Qmax, PVR, TPV, and PSA were used as the input variables. They reported that overall sensitivity and specificity were 71% and 69%. Wadie et al. [15] reported the higher predictive power of ANN compared to conventional statistical models among 460 subjects using only IPSS. However, the same group presented another ANN model considering average flow rate and Qmax on UFM, PVR, and TPV in variable conditions and showing only moderate performance with 76% accuracy [17]. Another study reported 82% and 77% sensitivity and specificity, respectively, using IPSS, TPV, PSA, and UFM results [16]. Comprehensive results show, however, that the predictive performance of ANN is not superior to that of the conventional linear models. Moreover, due to the ‘black box’ nature of ANN, the model cannot be easily interpreted [18]. Therefore, these models do not explain the relative contribution of non-invasive clinical parameters to urodynamic BOO.

In general, the advantage of CBNs is that they can identify conditional independence relationships and thus make it possible to confirm the only direct independent causes of the events. We expected that this advantage of the CBN model could confirm independent variables for the prediction of BOO. In this study, through the established CBN model, we found that TPV, Qmax, and PVR were important predictors of BOO (Fig. 1). Our BOO prediction model with only three independent variables (TPV, Qmax, and PVR) showed moderate predictive value (Table 2)

To compare the performance of the BOO prediction model using the three selected independent predictors, the LR model was established from the same dataset and the predictive powers of the two models were compared (Table 2). The LR model showed a predictive performance comparable with that reported in previous studies [10]–[13]. The predictive performance of the CBN model was statistically inferior to that of the LR model (AUROC: 0.772 vs. 0.798; p = 0.020). However, when only the three variables (TPV, Qmax, and PVR) were taken into account to predict BOO, the accuracy was not overly compromised compared with when using the complex equations (considering age, Qmax, PVR, TPV, TZV, PSA, IPSS item 2, and item 4 as predictive variables), which were derived from the LR model. These three non-invasive clinical parameters are also routinely evaluated in actual clinical practice for BPH patients.

Indeed, our CBN model comprised categorized values of clinical parameters due to in nature characteristics of CBN model for clarifying interactions between the variables. It is not clear whether the lower performance of CBN originates from the elliptical non-invasive clinical parameters or from transforming the clinical parameters into categorical variables to estimate the CBN models. However, it is interesting that the BOO can be predicted moderately with only three non-invasive clinical parameters. This study was unable to conclude whether the other variables that show conditional independence can be excluded for BOO prediction.

Our data showed that TPV was the most important predictive factor for BOO (R = 0.391), followed by, Qmax (R = −0.253), and PVR (0.214) in that order. Our results are consistent with those of previous studies which reported that TPV had a higher correlation with BOO compared to the other non-invasive clinical parameters [10]–[13]. These results suggest that TPV is the most important clinical parameter for BOO prediction in real clinical practice and that TZV and PSA do not need to be considered as predictors.

Qmax and PVR also had a moderate correlation with BOO(|R| range: 0.214–0.253). CBN showed that these variables are independent predictors of BOO. Therefore, these clinical parameters should be considered in BOO prediction. Previous studies considered various combinations of UFM results, such as Qmax, average flow rate (Qavg), and PVR in prediction models [9]–[13], but it has not yet been concluded which variables are the more important predictors of BOO. Our CBN model showed that Qmax and PVR are important for BOO prediction.

It is interesting that all IPSS items showed conditional independence (not independent predictors) for BOO prediction (Fig. 1). Previous studies excluded the IPSS from the BOO prediction model [10]–[13], and van Venrooij et al. [4] reported that IPSS has no statistical correlation with urodynamic obstruction grade; these are in agreement with our CBN results. However, in our LR model IPSS item 2 (frequency) and IPSS item 4 (urgency) were predictors of BOO (p range: <0.001 to 0.007).

The strength of this study is that we made our non-missing dataset of 1,381 patients large enough to support the construction of the CBN model. Moreover, in our study, all of the UDS were performed uniformly using the same protocol following ICS recommendations [22]. However, our current study has some limitations. Our model was unable to account for the weight of each independent predictor. Therefore, the relative importance of predictors should be identified by means of indirect correlation analysis. Second, our CBN model is built from a cross-sectional database; hence, in a strict sense, our model did not show cause-effect relationships between parameters, but showed simple correlations or interactions. It is thus impossible to confirm variables that precede the cause. Finally, the predictive power of CBN model was too low for the model to be considered to be useful in clinical practice. We believe that additional well-designed and in-depth researches into the CBN model are needed.

## Conclusions

Our results show that TPV, Qmax, and PVR are independent non-invasive predictors of BOO. Among them, TPV is the most important clinical parameter for predict of BOO.

## Supporting Information

Appendix S1 **Banjo setting file used for causal Bayesian network model of this study.**
(TXT)
